# Expression characteristics, prognostic value, and immune-related analysis of PPP2R1A in lung adenocarcinoma

**DOI:** 10.3389/fimmu.2025.1652629

**Published:** 2025-12-02

**Authors:** Shanshan Xiao, Yuhong Hu, Yawen Li, Xin Zhang, Zijun Xiao, Mingyou Dong, Lusheng Liao

**Affiliations:** 1 Obstetrics and Gynecology Department, Affiliated Hospital of Youjiang Medical University for Nationalities, Baise, Guangxi, China; 2 Guangxi Engineering Research Center for Precise Genetic Testing of Long-dwelling Nationalities, Youjiang Medical University for Nationalities, Baise, Guangxi, China; 3 Guangxi Engineering Research Center for Precise Genetic Testing of Long-dwelling Nationalities, Affiliated Hospital of Youjiang Medical University for Nationalities, Baise, Guangxi, China; 4 Key Laboratory of Research on Environment and Population Health in Aluminum Mining Areas, Education Department of Guangxi Zhuang Autonomous Region, Baise, Guangxi, China

**Keywords:** lung adenocarcinoma, PPP2R1A, tumor microenvironment, immune infiltration, Kaplan-Meier plotter

## Abstract

**Introduction:**

Lung adenocarcinoma (LUAD) is a major subtype of lung cancer with poor prognosis. The protein phosphatase 2 regulatory subunit A alpha (PPP2R1A) plays complex roles in tumorigenesis, but its function and clinical significance in LUAD remain unclear.

**Methods:**

We analyzed PPP2R1A expression across cancers using the Xiantao Academic Online tool and TCGA data. Diagnostic potential was evaluated via ROC curve analysis. Prognostic value was assessed using Kaplan-Meier survival and Cox regression analyses. Protein-protein interaction networks and functional enrichment analyses were conducted to explore molecular mechanisms. Immune infiltration patterns were investigated using TIMER2.0. Experimental validation was performed through CRISPR/Cas9-mediated knockdown in A549 cells, followed by functional assays including CCK-8, clonogenic, wound healing, and Transwell assays.

**Results:**

PPP2R1A was significantly upregulated in LUAD tissues compared to normal controls (P < 0.05). It demonstrated modest diagnostic value with an AUC of 0.593. High PPP2R1A expression was associated with poor progression-free survival (FP) and overall survival (OS), particularly in early-stage disease. PPP2R1A expression correlated with advanced N stage and tumor stage. Functional enrichment analysis revealed involvement in protein dephosphorylation, cell cycle regulation, and metabolic pathways. Immune infiltration analysis showed significant correlations with macrophage and CD4+ T cell infiltration. Experimental validation confirmed that PPP2R1A knockdown significantly inhibited LUAD cell proliferation, migration, and invasion (P < 0.01).

**Discussion:**

PPP2R1A is overexpressed in LUAD and associated with poor prognosis, potentially serving as an oncogene by regulating key signaling pathways and immune microenvironment. Its knockdown suppresses malignant phenotypes, highlighting its potential as both a prognostic biomarker and therapeutic target in LUAD.

## Introduction

Lung adenocarcinoma (LUAD), the most prevalent histological subtype of non-small cell lung cancer (NSCLC), remains a leading cause of cancer-related mortality worldwide, accounting for approximately 40% of all lung cancer cases (1, 2). Despite therapeutic advances, LUAD maintains high morbidity and mortality rates due to its marked tumor heterogeneity and frequent development of drug resistance (1, 3). Current treatment modalities including surgery, radiotherapy, and molecular targeted therapies have improved clinical prognosis, yet long-term survival rates remain unsatisfactory (2, 4). Major therapeutic challenges stem from the disease’s cellular complexity and the limited efficacy of existing treatments across diverse patient populations (3, 4). Early diagnosis is particularly difficult as LUAD patients often present with inapparent symptoms during initial stages (5, 6). Conventional diagnostic methods relying on imaging and pathological examination lack sufficient accuracy, creating an urgent need for reliable early diagnostic biomarkers (7, 8). The identification of novel prognostic biomarkers and therapeutic targets is crucial for improving risk stratification and enabling personalized treatment approaches (9, 10). Recent studies highlight the potential of molecular biomarkers derived from bioinformatics analyses to enhance early detection accuracy and predict treatment outcomes (10–13). These developments may address current diagnostic limitations and ultimately improve patient survival (14, 15).

The protein phosphatase 2 regulatory subunit A alpha (PPP2R1A) is a critical scaffold protein of the PP2A complex, which plays a dual role in tumorigenesis depending on cellular context. While PPP2R1A is recognized as a tumor suppressor in certain cancers, emerging evidence suggests its potential oncogenic properties in gastric cancer (GC), where its silencing significantly inhibits proliferation, migration, and invasion while promoting apoptosis in GC cells (*P* < 0.01) (16). Notably, elevated PPP2R1A expression correlates with poorer 5-year survival in GC patients (*P* < 0.001) (16), suggesting its prognostic value. In uterine cancer, recurrent PPP2R1A mutations (P179R and S256F) drive tumorigenesis and metastasis (17), while in colorectal cancer, PPP2R1B (a paralog) inactivation promotes liver metastasis through ERK pathway activation (18). Despite these findings in various malignancies, the role of PPP2R1A in lung adenocarcinoma (LUAD) remains poorly characterized compared to other PP2A subunits like PPP2R2A, which regulates EMT in NSCLC (19). This knowledge gap highlights the need to investigate PPP2R1A’s function in LUAD, particularly given PP2A’s established role as a negative regulator of oncogenic pathways (20) and the frequent dysregulation of protein phosphatases in lung cancer pathogenesis. Further exploration of PPP2R1A in LUAD could reveal novel mechanisms of tumor progression and therapeutic vulnerabilities.

In this study, we conducted a comprehensive analysis of the PPP2R1A gene expression in lung adenocarcinoma (LUAD), exploring its role in tumor progression, prognosis, and immune modulation. We utilized various bioinformatics tools to assess the expression of PPP2R1A across multiple cancer types, revealing its upregulation in LUAD. Additionally, we investigated its diagnostic potential and found modest discriminative capacity. The study also examined the association between PPP2R1A expression and clinicopathological features, such as tumor stage and lymph node involvement, highlighting its prognostic significance in early-stage LUAD. Furthermore, we explored the genetic interactions and immune cell infiltration patterns related to PPP2R1A, revealing its involvement in critical pathways associated with tumor progression. Experimental validation confirmed that PPP2R1A knockdown inhibited proliferation, migration and invasion, supporting its role in LUAD progression and highlighting its potential as a biomarker for diagnosis and prognosis. This could provide a foundation for future therapeutic strategies targeting PPP2R1A to modulate tumor growth and immune responses in LUAD patients.

## Materials and methods

### Differential expression and clinical correlates of PPP2R1A in LUAD

This study utilized the Xiantao Academic platform (https://www.xiantaozi.com/) (21) to conduct differential expression and clinical correlation analyses of PPP2R1A in LUAD. Data associated with 598 mRNA LUAD samples, along with 59 matched adjacent normal tissue samples, all acquired from The Cancer Genome Atlas (TCGA database https://tcga-data.nci.nih.gov/). The mRNA expression data from TCGA were normalized as Transcripts Per Million (TPM). The parameters for differential expression analysis were set as follows: 1) tumor type selected as ‘LUAD’, transcriptomic data format selected as ‘TPM’, and other parameters set to default values. Clinical and pathological variables encompassed Gender, T stage, N stage, M stage, and overall stage classification. As all data utilized in this study were sourced exclusively from the publicly available TCGA database, ethics committee or institutional review board approval was not required. The analysis strictly adhered to TCGA publication guidelines.

### Genetic alteration analysis

In this study, utilizing the cBioPortal platform (https://www.cbioportal.org/) (22), we interrogated alteration frequency, copy number variations, and mutation types of PPP2R1A across malignancies within the TCGA pan-cancer atlas, defining its comprehensive genetic alteration landscape.

### Co-expression network analysis and key gene validation

Using the pan-cancer analysis platform GEPIA2 (http://gepia2.cancer-pku.cn/) (23), we systematically retrieved and downloaded the top 100 gene lists with significant co-expression relationships with the PPP2R1A in lung adenocarcinoma. Using the built-in ‘correlation analysis’ module in GEPIA2, the top three most highly correlated genes in the co-expression network were validated using scatter plots based on TCGA tumor data.

### Tumor immune assessment resources

Systemic immune microenvironment profiling was performed by using the TIMER 2.0 tool (http://timer.cistrome.org/) (24, 25). The platform integrates TCGA pan-cancer data encompassing transcriptomes of 16,283 tumor samples (representing 11,300 patients) with immune cell infiltration estimates across 33 malignancies. We leveraged two analytical modules: (1) the ‘timer-Gene’ module was used to assess PPP2R1A expression correlations with immune infiltration levels specifically in lung adenocarcinoma (LUAD), and (2) the ‘Immune-gene’ module was used to evaluate PPP2R1A-immune infiltration associations in the TCGA cohort.

### Protein interaction analysis

In this study, we constructed the protein interaction network of PPP2R1A using the STRING database (https://string-db.org/) (26). The specific workflow is as follows: first, gene name input: PPP2R1A, Species selection: Homo sapiens. The interaction sources were selected as ‘text mining and experiments’, interaction thresholds were defined as follows: (1) minimum interaction score = 0.150 (low confidence cutoff), and (2) maximum interactor displays limit = 50 proteins.’ The remaining parameters were set to the system default configurations.

### Survival and prognosis analysis

Prognostic significance of PPP2R1A in lung adenocarcinoma was interrogated via the Kaplan-Meier Plotter (https://kmplot.com/) (27). Patients were stratified into high- and low-expression groups by median expression of PPP2R1A, the relationship between PPP2R1A expression and Failure-free survival (FP), Overall survival (OS), and Post-progression survival (PPS) was subsequently analyzed to explore the prognostic value of PPP2R1A in lung adenocarcinoma. Statistical significance was assessed using the log-rank test, and hazard ratios (HR) with 95% confidence intervals (CI) were calculated.

### Integrating mass spectrometry proteomics and transcriptome data

Utilize CPTAC (https://cptac-data-portal.georgetown.edu/) The “Protein Expression Analysis with cProstite” module of the database systematically analyzes the expression characteristics of PPP2R1A protein in LUAD. By integrating high-depth mass spectrometry proteomics and matching transcriptome data, the relative protein abundance and mRNA expression levels of PPP2R1A in tumor tissue and adjacent non tumor tissue were quantitatively compared, and its clinical significance was further evaluated. To more clearly illustrate the relationship between PPP2R1A protein abundance and mRNA expression levels, we have included a scatter plot depicting the protein abundance and mRNA expression levels in tumor and adjacent non-tumor tissues. We have also calculated the correlation between the two. The proteomic data were normalized as log_2_ (TMT ratio), with median centering across samples, and the accompanying mRNA data were normalized as Counts Per Million (CPM), followed by log_2_ transformation.

### Cell culture

The A549 lung adenocarcinoma cell line used in this study (28, 29) was obtained from the Shanghai Cell Bank, Chinese Academy of Sciences. Prior to subsequent experiments, a subpopulation of A549 cells was subjected to CRISPR/Cas9-mediated PPP2R1A gene knockdown to establish a gene-modified cell model, which was used for comparative analysis with the parental wild-type A549 cells. To ensure the reliability of the gene-modified model, the knockdown efficiency was verified by quantitative real-time polymerase chain reaction (qRT-PCR) and Western blotting. Importantly, this PPP2R1A knockdown was confirmed to be stable; the modified cells were continuously passaged and cultured, and the persistent downregulation of PPP2R1A expression was monitored and validated for at least 10 consecutive passages to exclude the possibility of gene expression recovery during long-term culture.

Cells (both wild-type and stable PPP2R1A-knockdown A549 cells) were cultured in a humidified incubator maintained at 37 °C with 5% CO_2_. The culture medium consisted of high-glucose Dulbecco’s Modified Eagle Medium (DMEM) (Gibco, C11995500BT), supplemented with 10% fetal bovine serum (Oricell, FBSSR-01021) and 1% penicillin-streptomycin (Gibco, 15140122). All cell-based assays were performed with triplicate replicates.

### Total RNA extraction and qRT-PCR analysis

Log-phase cells were harvested for total RNA extraction using TRIzol reagent (SparkZol Reagent, AC0101-B). cDNA synthesis was then performed using reverse transcription reagents (Tolobio, 22107). After the addition of the corresponding primers, cDNA, and fluorescent dye (Tolobio, 22204-1), the reaction mixture was transferred to an eight-well plate and analyzed using the Roche LightCycler 96 system to quantify the expression levels of target genes. Gene expression fold changes for each triplicate sample set were calculated using the 2^−ΔΔCT^ method, with normalization against GAPDH. The primer sequences used were: PPP2R1A-F: CGGAGACCCCAACTACCTGC; PPP2R1A-R: GCCACATTGAAGCGGACATT.

### Cell proliferation assay

Cell proliferative potential in lung adenocarcinoma was assessed using two complementary approaches: CCK-8 viability assays and clonogenic formation assays. Log-phase cells were trypsinized (25200056, Gibco) and plated at 300 cells per well in six-well plates (140675, Thermo). For the clonogenic assay, cells were cultured for 14 days, allowing colonies to form into morphologically distinguishable clusters. The six-well plates were washed twice with PBS (Gibco, C10010500BT) to remove suspended cells and residual substances. Cells were then fixed with 4% paraformaldehyde (Biosharp, BL539A) for 30 minutes, followed by staining with crystal violet (Solarbio, G1063) for 10 minutes. After drying, images were captured, and the stained colonies were counted and statistically analyzed.

For the CCK-8 assay, 2,000 cells in the exponential growth phase were carefully seeded into each well of a 96-well plate (Thermo, 167008) to ensure uniformity and optimal conditions for subsequent analysis. Cells were cultured for 24, 48, 72, and 96 hours. Following incubation, 10 μL of CCK-8 reagent (HY-K0301-500T, MCE) was added to each well. Absorbance was measured at 450 nm using an INFINITE 200 PRO microplate reader (3005030301), and growth kinetic curves were generated.

### Cell scratch assay

The day before the experiment, logarithmic-phase A549 cells were trypsinized and resuspended as single-cell suspension, then seeded into 6-well plates at 3×10^5^ cells per well and cultured overnight until 90–100% confluence was reached. The cells covering the plate were removed, and 200 μL of autoclaved wall head solution was applied to draw a straight line perpendicular to the six-well plate. After rinsing with PBS, serum-free medium was added. Images were captured under a microscope (Axio Vert A1, 07060200), and the initial scratch area (A_0h_) was recorded. After 48 hours of culture, images were taken at the same location. The migration kinetics were assessed by calculating the wound closure percentage using the following formula: Closure rate = [(A_0h_ - A_48h_)/A_0h_] × 100, where A_x_ represents the wound area at X hours post-scratching.

### Cell invasion and migration experiment

To assess migratory and invasive capacities, Transwell assays were conducted. Log-phase cells were harvested and resuspended in serum-free medium at a concentration of 1 × 10^5^ cells/mL. The Transwell system (TCS003024, JET) was set up with 200 μL of serum-free cell suspension in the upper compartment and 600 μL of complete medium (10% serum) in the lower compartment. The system was then incubated at 37 °C for 24 hours to allow migration and invasion. After incubation, the chamber was removed and fixed with paraformaldehyde for 30 minutes, followed by staining with 0.5% crystal violet for 5 minutes. The chamber was rinsed with PBS, and excess cells were removed using a cotton swab. Multiple fields of view were captured under a microscope, and statistical analysis was performed. For the invasion assay, 100 μL of matrix gel (Corning, 356234) was added to the Transwell insert before the experiment, allowed to solidify in a 37 °C incubator, and then the subsequent steps were performed.

### Statistical analysis

All statistical analyses in this study were performed using SPSS statistical software (version 26.0; IBM, NY, USA). Data were presented as mean ± standard deviation (Mean ± SD) or median (Median) and interquartile range (IQR). Intergroup comparisons were statistically evaluated using either the Student’s *t*-test or the Mann-Whitney U test, with the selection contingent upon the underlying data distribution, while for comparing multiple groups, one-way analysis of variance (ANOVA) or Kruskal Wallis test is used. To evaluate the prognostic significance of PPP2R1A in lung adenocarcinoma (LUAD), Kaplan-Meier survival curves were generated with subsequent intergroup comparisons conducted via log-rank testing. Hazard ratios (HR) and 95% confidence intervals (CI) were derived from the Cox model. To systematically explore the potential association between PPP2R1A expression levels, clinical characteristics, and patient survival prognosis, used Cox proportional hazards regression model to perform multivariate survival analysis on survival outcome data. Differential expression analysis of PPP2R1A was performed via the Xiantao platform using TCGA-derived LUAD and matched normal cohorts, with expression disparities quantified by distribution-appropriate statistical tests (Student’s *t*-test or Mann-Whitney *U* test). Correlation analysis was performed to calculate the correlation between PPP2R1A and other genes using the Spearman correlation coefficient, employing either the Pearson or Spearman method depending on the distribution characteristics of the data. For multiple comparisons in subgroup analyses, False Discovery Rate (FDR) correction was applied where appropriate. Statistical significance was established at *P* < 0.05, with a more stringent threshold of *P* < 0.001.

## Results

### PPP2R1A gene expression analysis in lung adenocarcinoma

**Figure 1:**
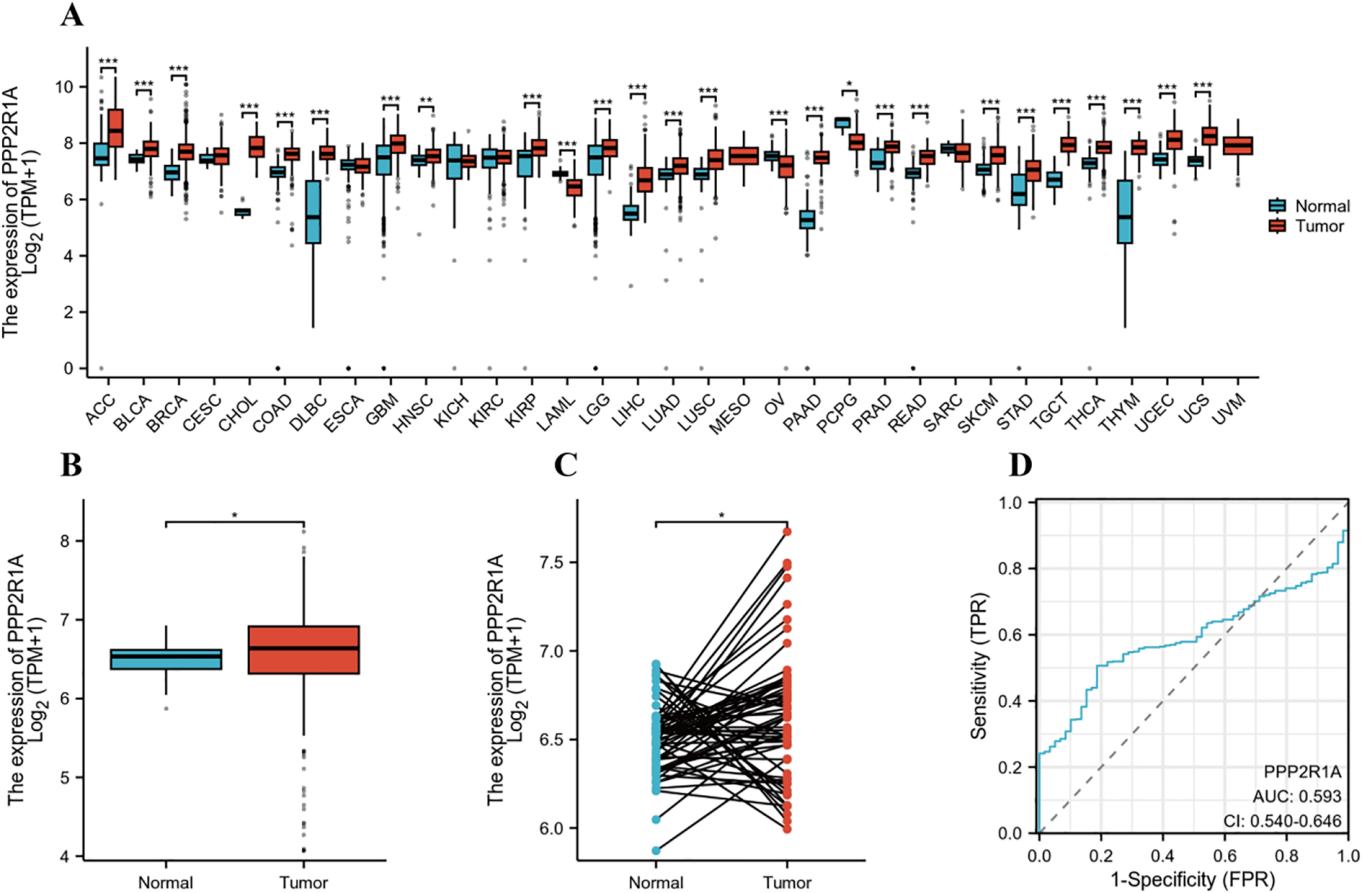
PPP2R1A gene expression profile in lung adenocarcinoma (LUAD). **(A)** Comprehensive pan-cancer analysis of PPP2R1A expression across malignancies using integrated TCGA and GTEx datasets. **(B)** Comparative analysis of PPP2R1A transcript abundance between LUAD tumors and matched non-neoplastic counterparts in TCGA cohort. **(C)** Validation of differential expression in 58 paired LUAD tissues versus histologically normal adjacent specimens from TCGA database. **(D)** Diagnostic potential evaluation for PPP2R1A in LUAD based on ROC curve analysis. *p < 0.05, **p < 0.01, ***p < 0.001.

Using the Xiantao online tool to assess the expression levels of PPP2R1A mRNA, we detected a significant increase in PPP2R1A expression in 26 cancers (**Figure 1A**). Unpaired analysis results showed a significant upregulation of PPP2R1A in lung adenocarcinoma. (*P* < 0.05) (**Figure 1B**). Paired analysis results were consistent with unpaired results, further confirming that PPP2R1A expression levels were significantly increased in lung adenocarcinoma samples (*P* < 0.05) (**Figure 1C**). Subsequently, Further assessment of PPP2R1A’s diagnostic potency in lung adenocarcinoma yielded an AUC of 0.593 (95% *CI*: 0.54-0.64), indicating modest discriminative capacity for malignant identification (**Figure 1D**). Notably, analysis of the independent CPTAC dataset revealed a contrasting pattern, with both PPP2R1A protein (TMT log_2_ ratio) and mRNA (gene expression count) levels being significantly lower in LUAD tumor tissues compared to adjacent normal tissues (*P* < 0.001; **Supplementary Figures 1A, B**). Furthermore, the scatter plot in (**Supplementary Figure 1C**) illustrates the correlation between PPP2R1A mRNA and protein levels across tumor and adjacent normal tissues. The total correlation coefficient was -0.5090, indicating a moderate negative correlation between mRNA and protein levels. This discrepancy between TCGA and CPTAC datasets may stem from differences in sample cohorts, normalization methodologies, or post-transcriptional regulatory mechanisms.

### The prognostic value of PPP2R1A in lung adenocarcinoma

**Figure 2:**
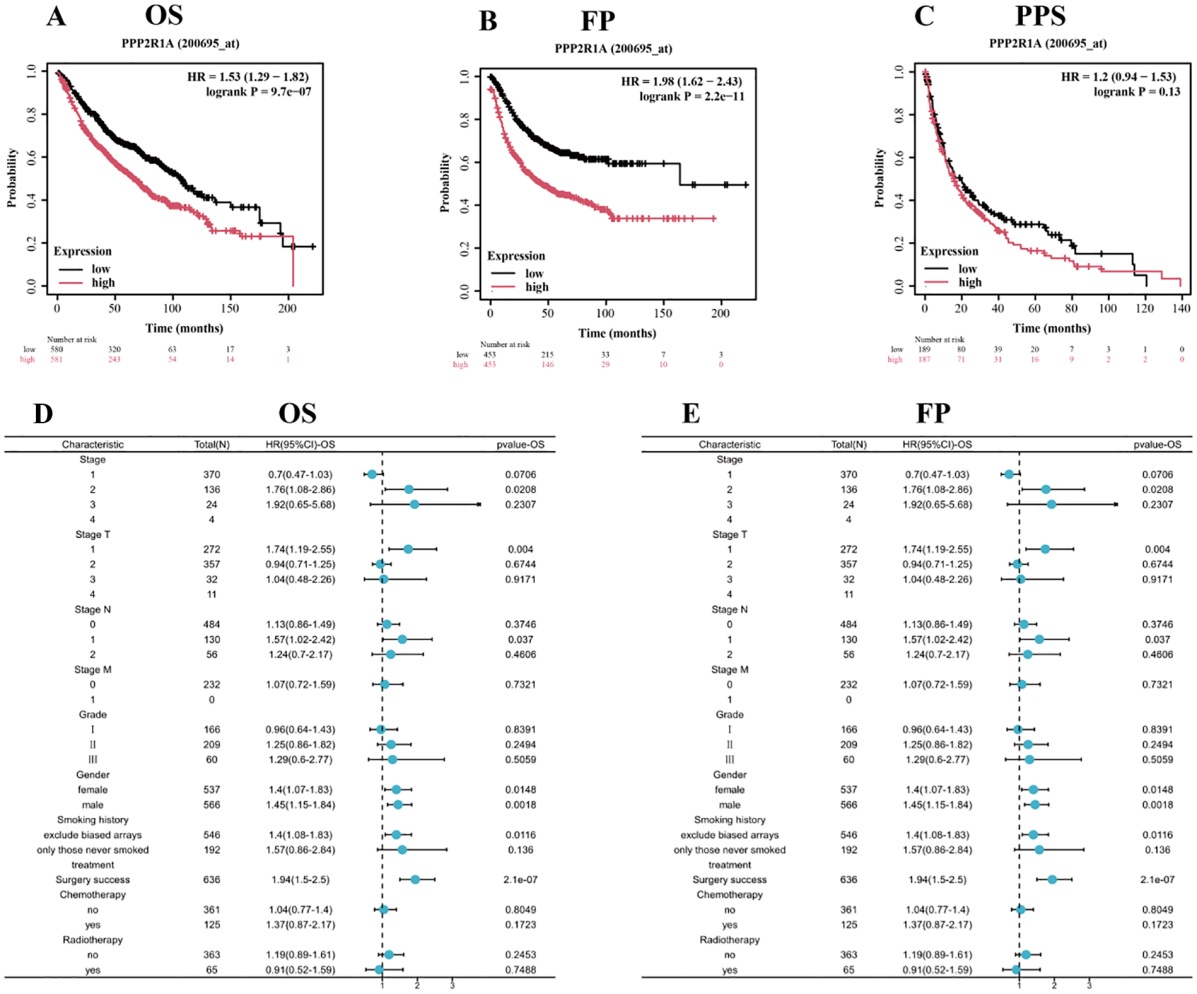
Prognostic significance of PPP2R1A expression in lung adenocarcinoma (LUAD). **(A-C)** Survival analysis delineating overall survival (OS), first progression (FP), and post-progression survival (PPS) probabilities through Kaplan-Meier curves stratified by PPP2R1A expression levels (log-rank *p* values annotated). **(D, E)** Multivariate Cox regression analysis visualizing the association between PPP2R1A transcript abundance and clinicopathological covariates via forest plots (*HR* with 95% *CI*).

We divided patients into high and low expression groups based on PPP2R1A expression levels. Kaplan-Meier (KM) survival analysis showed that PPP2R1A overexpression in patients with lung adenocarcinoma was significantly associated with poor FP and OS outcomes. Subsequently, we analyzed the relationship between PPP2R1A expression and adverse FP and OS outcomes in different clinical subgroups (**Figures 2A-C**). The results showed that patients with stage I LUAD exhibited the strongest PPP2R1A-related survival disadvantage, suggesting a higher carcinogenic impact in early disease (**Figures 2D, E**).

### Association between PPP2R1A expression and clinicopathological features of lung adenocarcinoma

**Figure 3:**
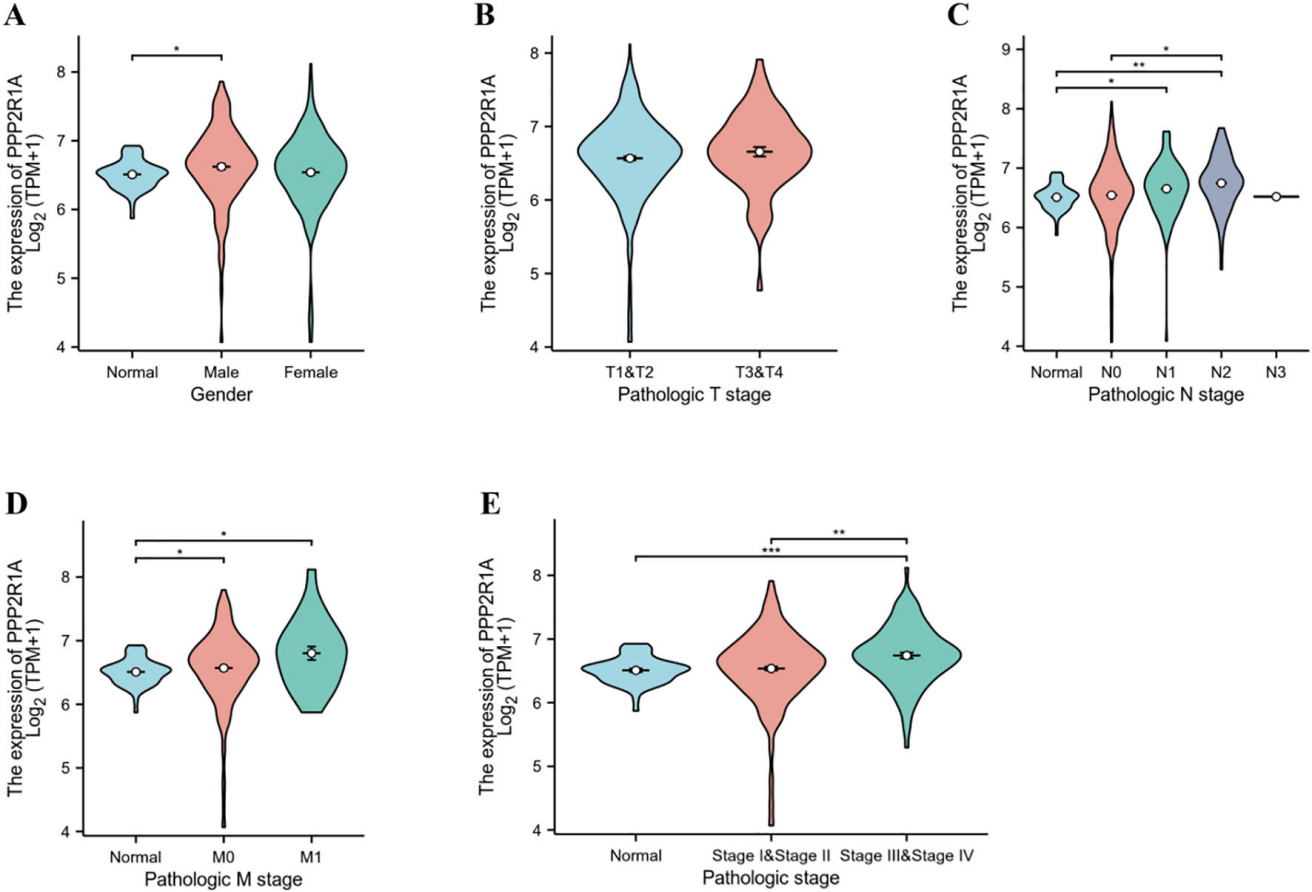
Association between PPP2R1A expression and clinicopathological features of lung adenocarcinoma. **(A)** Sex. **(B)** T stage. **(C)** N stage. **(D)** M stage. **(E)** pathologic stage. *p < 0.05, **p < 0.01, ***p < 0.001.

Subgroup analysis based on clinical pathological parameters further revealed the relationship between PPP2R1A and the clinicopathological features of lung adenocarcinoma. No statistically significant correlation was observed between PPP2R1A expression and either gender or T stage among lung adenocarcinoma patients (**Figures 3A, B**). The expression level of PPP2R1A was closely associated with N stage in lung adenocarcinoma patients, compared with N0 and N1 stage lung adenocarcinoma patients, PPP2R1A expression levels were significantly higher in N2 stage lung adenocarcinoma patients (**Figure 3C**). While PPP2R1A expression levels were higher in M1 stage lung adenocarcinoma patients, this difference did not reach statistical significance (**Figure 3D**). PPP2R1A expression was significantly associated with lung adenocarcinoma stage; advanced-stage (III-IV) LUAD patients exhibited significantly higher PPP2R1A expression compared to early-stage (I-II) cases (**Figure 3E**).

### Identification of PPP2R1A-associated genes and analysis of genetic variation characteristics in lung adenocarcinoma

**Figure 4:**
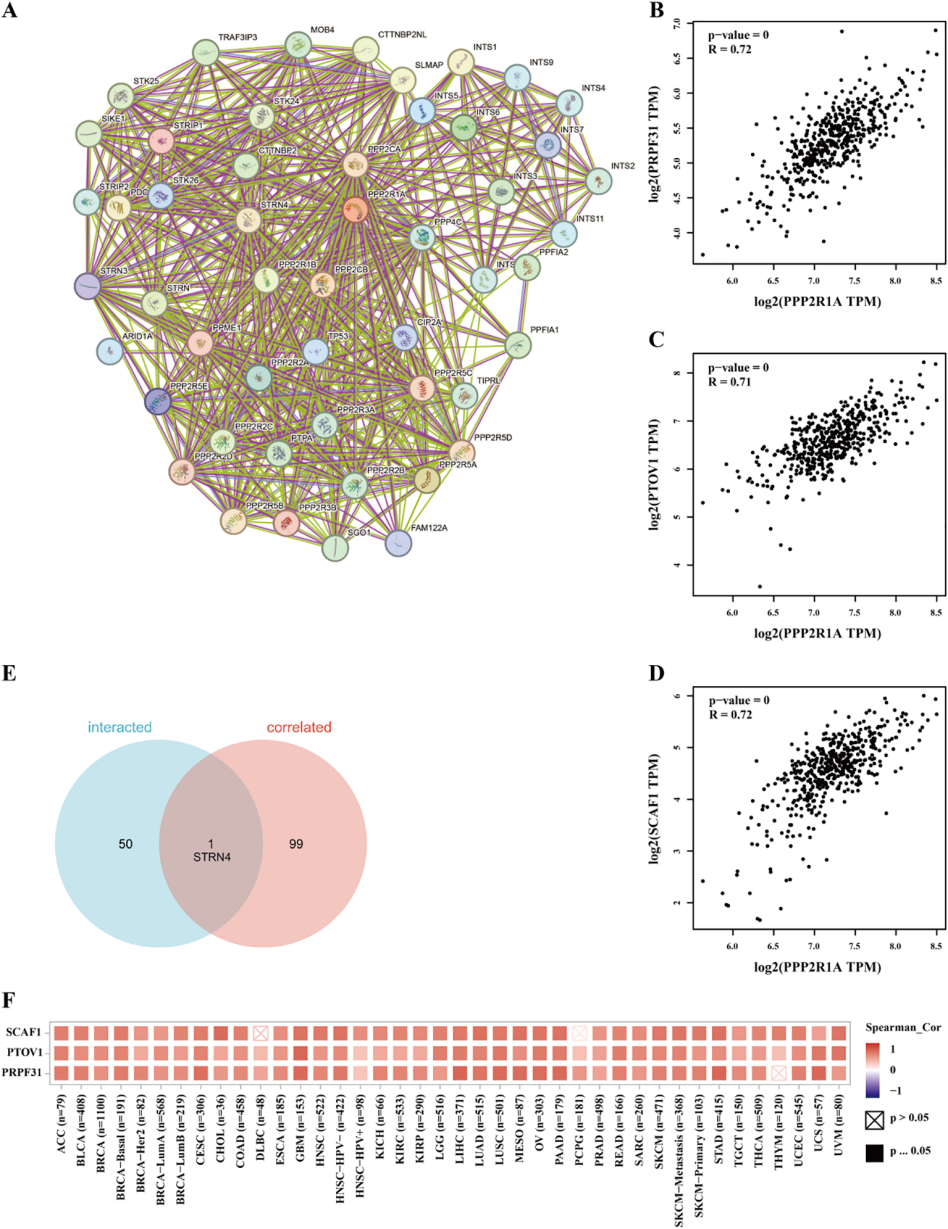
Identification of associated genes and analysis of genetic variation characteristics of PPP2R1A in lung adenocarcinoma. **(A)** Protein-protein interaction (PPI) network of PPP2R1A-binding partners reconstructed using STRING database. **(B-D)** Transcriptional covariation analysis demonstrating significant co-expression patterns between PPP2R1A and its top-correlated genes (PRPF31, PTOV1, SCAF1) derived from the top 100 PPP2R1A-associated genes in TCGA via GEPIA2 platform (Pearson’s r values indicated). **(E)** Consensus gene identification through Venn intersection of protein-interacting and co-expressed gene sets. **(F)** Pan-cancer co-expression profiling visualized by hierarchical clustering heatmap across multiple malignancies in TCGA cohort.

**Figure 5:**
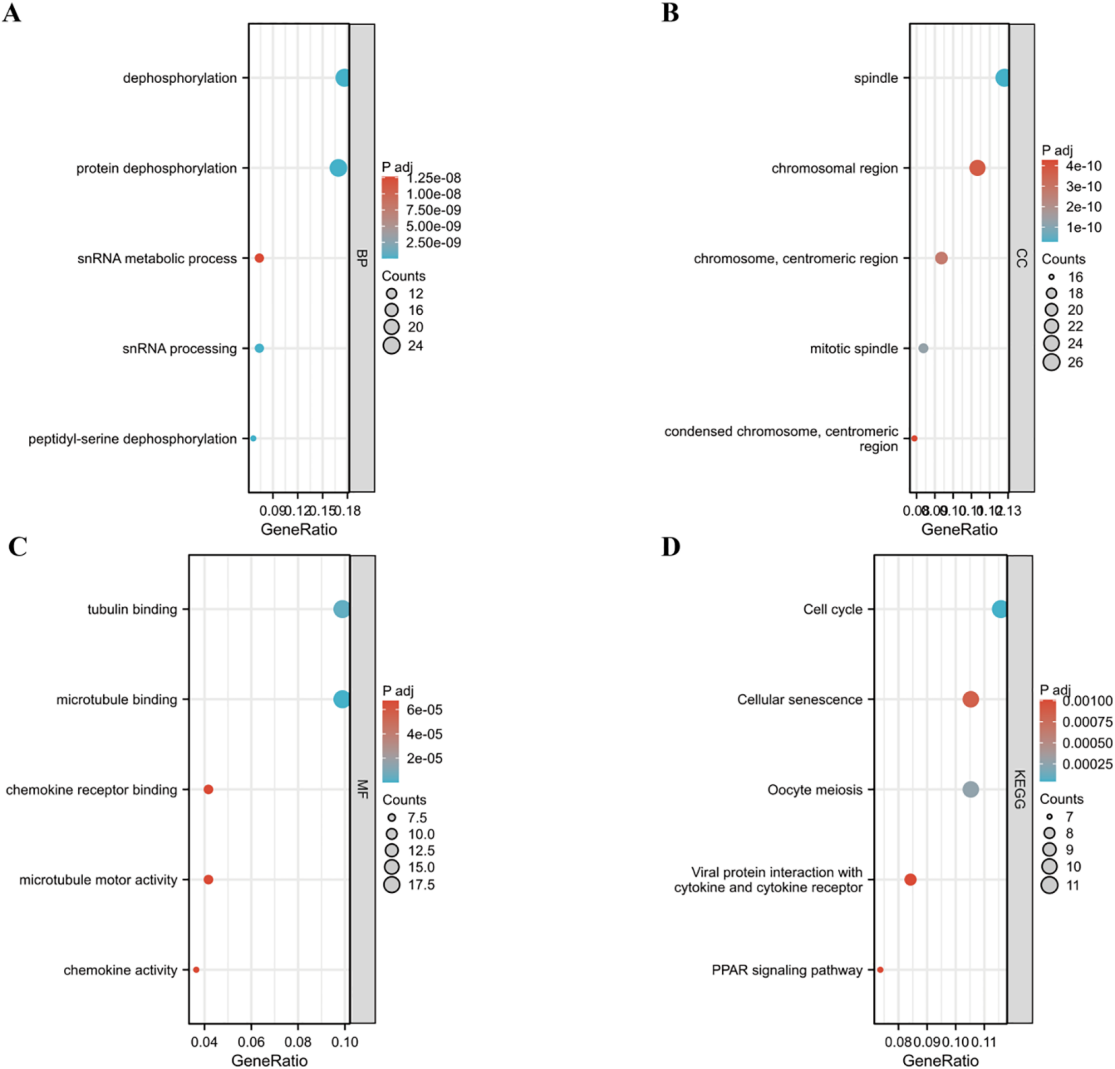
Functional enrichment profiling of PPP2R1A-associated gene signatures. **(A-C)** Systematic annotation of 150 PPP2R1A-interacting/correlated genes through Gene Ontology (GO) enrichment analysis encompassing. Biological processes (BP), Cellular components (CC), Molecular functions (MF). **(D)** Pathway-centric analysis via KEGG database.

**Figure 6:**
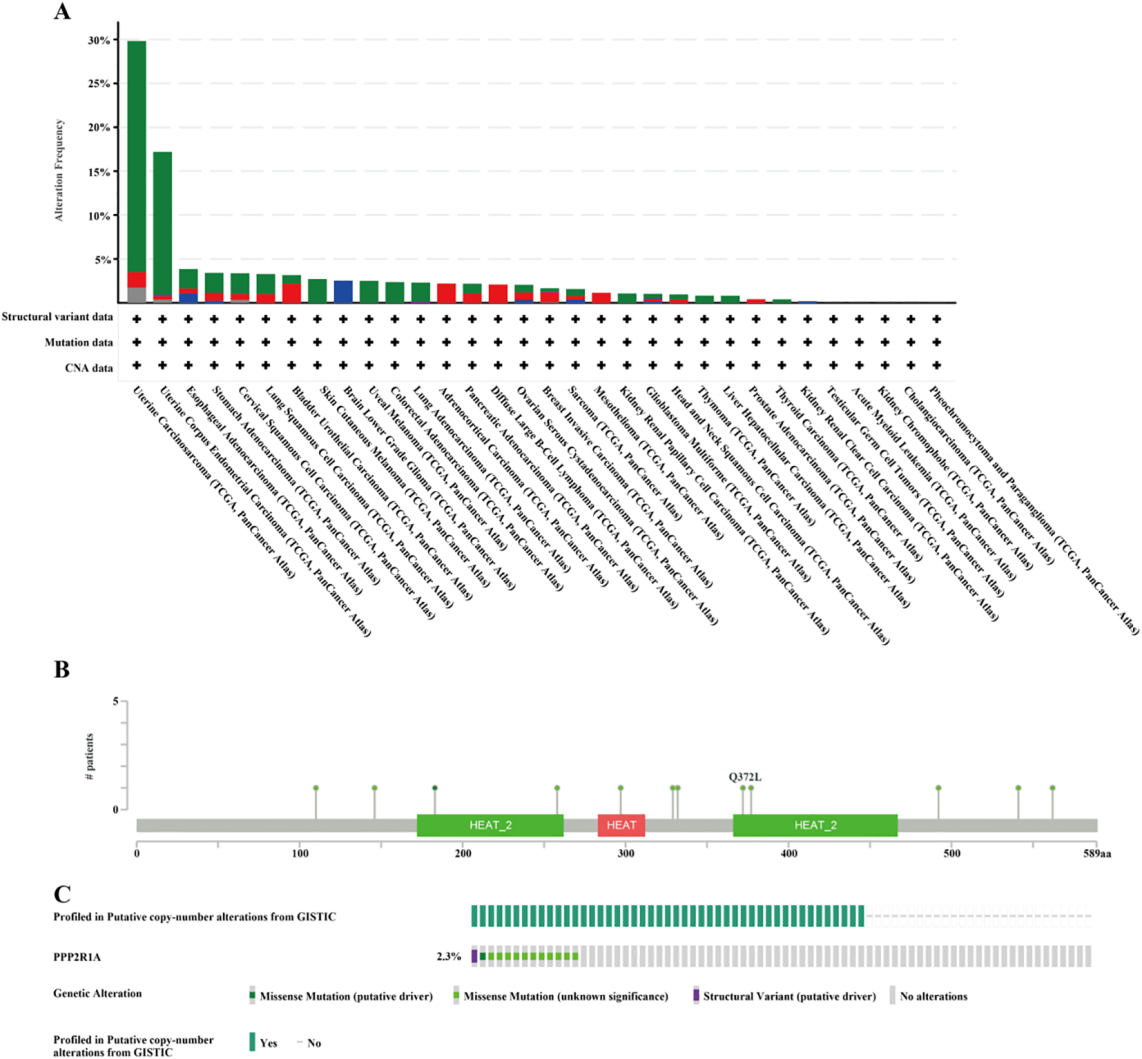
Mutation characteristics of PPP2R1A in lung adenocarcinoma using cBioPortal database. **(A)** PPP2R1A gene mutations in Pan cancer. **(B, C)** Mutation type and mutation rate of PPP2R1A gene in lung adenocarcinoma.

A protein-protein interaction (PPI) network for PPP2R1A was constructed through the STRING database, revealing close interactions with members of the protein phosphatase complex (e.g., PPP2CA, PPP2R2A) and INTS family proteins (e.g., INTS6, INTS1) (**Figure 4A**). In the TCGA lung adenocarcinoma cohort, PPP2R1A expression demonstrated significant positively correlated with PRPF31 (R = 0.72), SCAF1 (R = 0.72), and PTOV1 (R = 0.71) (**Figures 4B-D**). A Venn diagram shows that there is one common gene, STRN4, shared between the interacting genes and the expression-related genes (**Figure 4E**). In different tumors, PPP2R1A was significantly positively correlated with PRPF31, PTOV1, and SCAF1 (**Figure 4F**). To predict the functions of PPP2R1A-related genes, we performed Gene Ontology (GO) functional enrichment analysis on 150 PPP2R1A-related genes obtained from screening. Gene Ontology (GO) enrichment analysis revealed that the three most enriched GO terms in the three processes (BP, CC, MF) were ‘peptide serine dephosphorylation,’ ‘chromosome centromere region,’ and ‘chemokine receptor binding,’ indicating that PPP2R1A regulates protein dephosphorylation, cell cycle, and metabolic pathways, and synergistically drives lung adenocarcinoma progression with key genes such as PRPF31 and SCAF1 (**Figures 5A-C**). KEGG pathway enrichment analysis revealed that key pathways involving PPP2R1A-associated genes include ‘cell cycle,’ ‘cell senescence,’ and ‘PPAR signaling pathway,’ suggesting that PPP2R1A promotes tumor progression by regulating proliferation and metabolism (**Figure 5D**). Finally, we used the cBioPortal platform to investigate the genetic mutation characteristics of PPP2R1A in lung adenocarcinoma. Based on TCGA-LUAD data, the mutation frequency of PPP2R1A in lung adenocarcinoma was only 2.3%, primarily consisting of missense mutations in the phosphatase domain (e.g., Q372L) (**Figures 6A-C**).

### Immune cell infiltration analysis of PPP2R1A in lung adenocarcinoma

**Figure 7:**
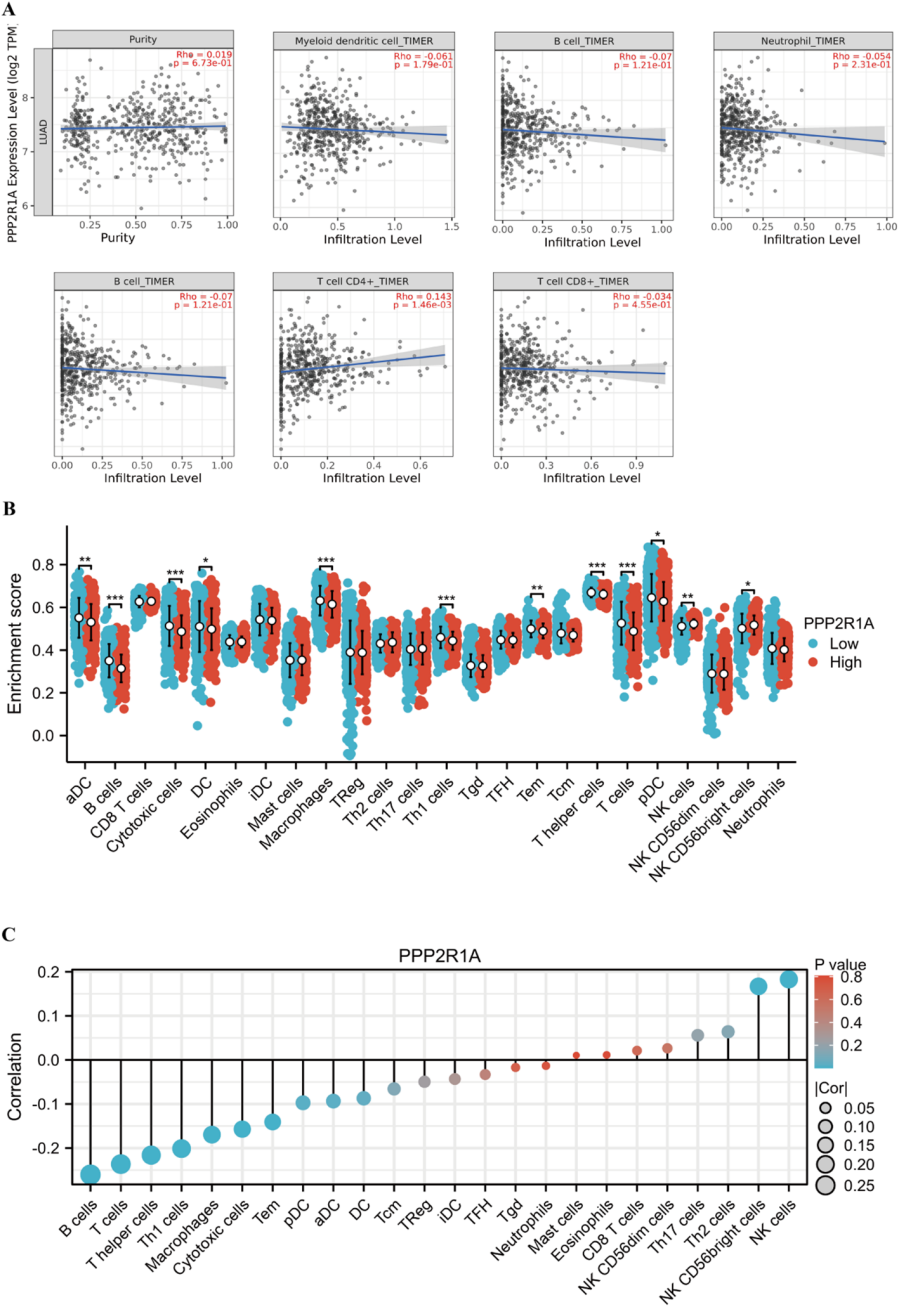
Multidimensional associations between PPP2R1A expression and tumor immune microenvironment in LUAD. **(A)** The correlation between PPP2R1A expression, tumor purity, and the infiltration levels of six immune cell types was analyzed using the TIMER database. **(B)** The difference in the infiltration levels of 24 immune cell types between the high expression and low expression groups of PPP2R1A. **(C)** The correlation between PPP2R1A expression and the infiltration levels of 24 immune cell types. *p < 0.05, **p < 0.01, ***p < 0.001.

To investigate PPP2R1A’s immunomodulatory potential, we assessed its co-expression patterns with tumor-infiltrating immune cell signatures across the LUAD. Immunoinfiltration analysis revealed PP2R1A showed significant positive correlations with macrophage and CD4 T cells infiltration (*P* < 0.05), but not significantly correlated with the infiltration level of myeloid dendritic cells, neutrophils, B lymphocytes and CD8 T cells (**Figure 7A**). To further characterize the macrophage polarization state associated with PPP2R1A, we analyzed its correlation with canonical M1 (NOS2, IL6, TNF, CD86, CD80) and M2 (TGFB1, CD163, MRC1, ARG1, IL10) macrophage markers. Contrary to our initial hypothesis, PPP2R1A expression demonstrated a significant negative correlation with the majority of M1 markers (e.g., CD80: R = -0.194, P < 0.001; CD86: R = -0.149, P < 0.001). Notably, it also showed significant negative correlations with several key M2 markers, including IL10 (R = -0.181, P < 0.001) and ARG1 (R = -0.129, P < 0.01). A notable exception was a significant positive correlation with the M2-related cytokine TGFB1 (R = 0.216, P < 0.001) (**Supplementary Figures 2A, B**). This complex correlation pattern suggests that PPP2R1A is linked to a specific immunosuppressive context, potentially defined more by TGFB1 signaling than a classical M2 polarization.

Furthermore, we investigated the relationship between PPP2R1A and regulatory T cell (Treg) infiltration by examining key Treg signature genes (FOXP3, IL2RA, CCR8, CTLA4). Strikingly, PPP2R1A expression was significantly negatively correlated with the functional Treg markers CTLA4 (R = -0.230, P < 0.001) and CCR8 (R = -0.148, P < 0.001), as well as with IL2RA (CD25) (R = -0.104, P < 0.05), while showing no correlation with the master transcription factor FOXP3 (R = 0.001, P > 0.05) (**Supplementary Figure 2C**). This inverse relationship with critical Treg effector molecules suggests that high PPP2R1A expression may be associated with an altered, or potentially dysfunctional, Treg compartment within the LUAD tumor microenvironment.

**Figure 8:**
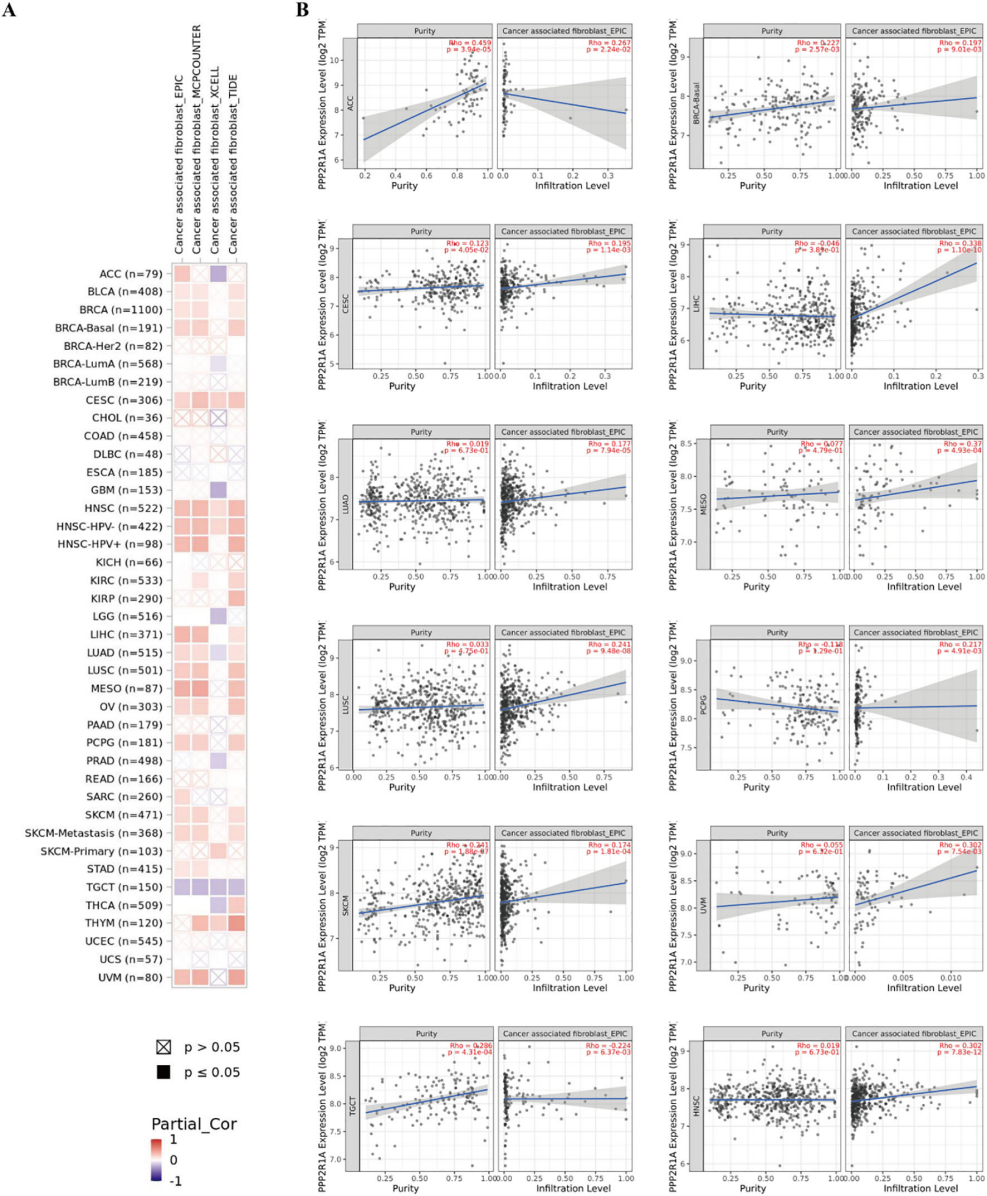
The correlation between PPP2R1A expression and tumor-associated fibroblasts in different cancers. **(A)** A correlation heatmap showing the relationship between PPP2R1A expression and tumor-associated fibroblasts in different cancers. **(B)** A scatter plot illustrating the correlation between PPP2R1A expression and tumor-associated fibroblasts.

Additionally, we investigated the relationship between PPP2R1A expression levels and the tumor immune microenvironment. First, we divided 598 patients into high and low expression groups according to the expression level of the PPP2R1A in the TCGA dataset. Subsequently, we performed comparative analysis of immune cell infiltration patterns between PPP2R1A-high and -low expression groups across 24 immune cell subtypes. In the high PPP2R1A expression group, sixteen cell types, including B cells, activated dendritic cells (ADC), cytotoxic cells, and T cells, were significantly elevated (**Figure 7B**). Additionally, sixteen of the 24 immune cell subtypes were negatively correlated with PPP2R1A expression (**Figure 7C**). Next, we employed TIMER2 to analyze the correlation between PPP2R1A gene expression patterns and immune infiltration levels across various tumor types in the TCGA. This analysis revealed consistent and statistically significant positive correlations between PPP2R1A expression and cancer-associated fibroblast (CAF) infiltration in multiple tumor types, including: head and neck squamous cell carcinoma, hepatocellular carcinoma, lung adenocarcinoma, malignant mesothelioma, and other tumor types (**Figure 8A**). Subsequently, a scatter plot generated using the most relevant algorithm presented the correlation between PPP2R1A gene expression and cancer-associated fibroblasts across diverse malignancies (**Figure 8B**).

**Figure 9:**
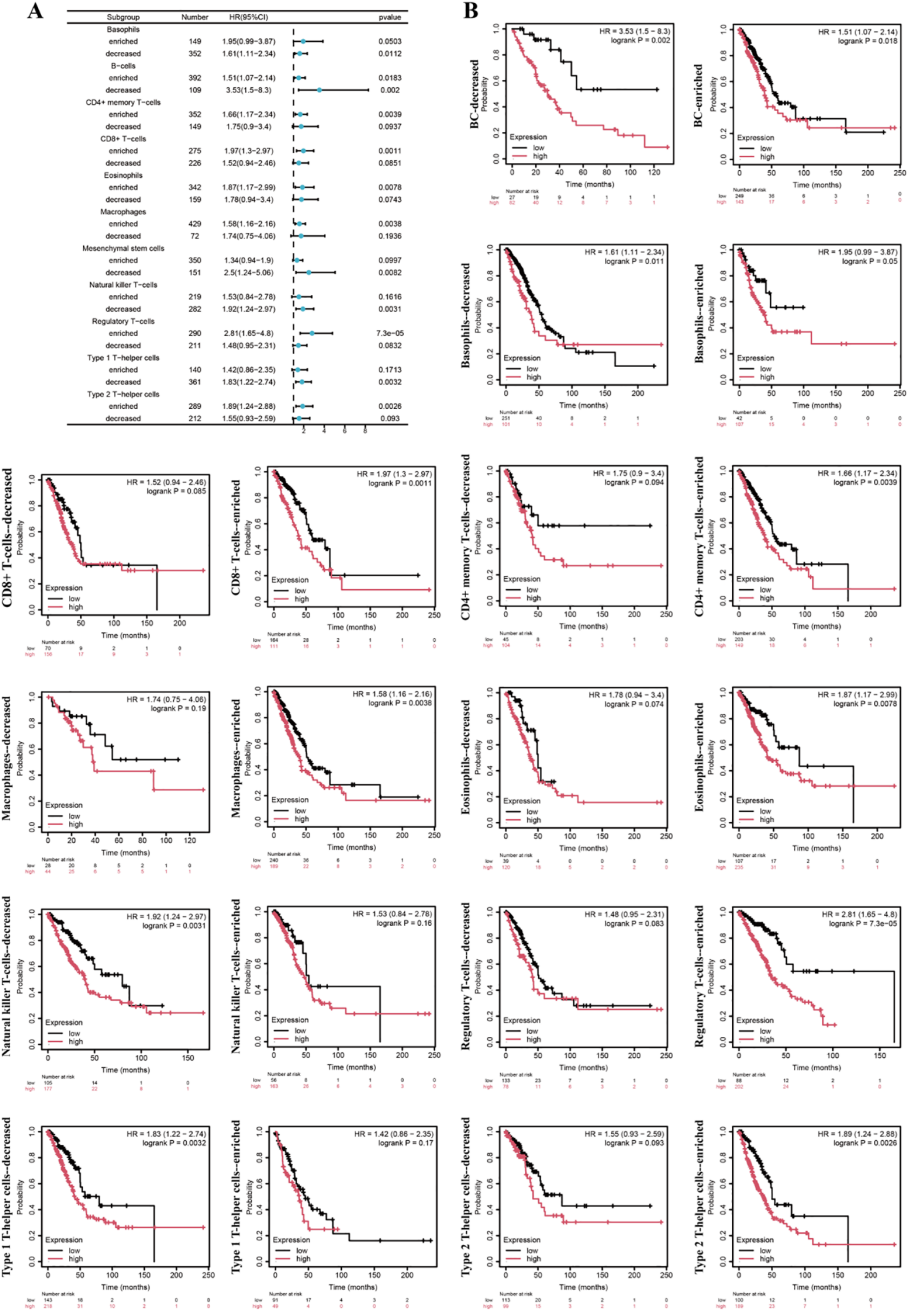
The association between different immune subgroups and prognosis of lung adenocarcinoma. **(A)** The forest plot. **(B)** Survival curve.

Hierarchical Cox regression analysis revealed the association between different immune cell subpopulations and prognostic features in lung adenocarcinoma patients (**Figures 9A, B**). The results showed that reduced mesenchymal stem cell levels and decreased natural killer T cells both demonstrating significant prognostic increased risk of death in lung adenocarcinoma patients. Conversely, the enrichment of regulatory T cells (Tregs) exhibited the strongest adverse prognostic impact, followed by CD8+ T cell infiltration. The prognostic dichotomy was particularly pronounced in T helper cell subpopulations: the depletion of type 1 T helper cells and the enrichment of type 2 T helper cells both demonstrated significant clinical implications.

### PPP2R1A knockdown inhibits the proliferation, invasion, and metastasis of lung adenocarcinoma

**Figure 10:**
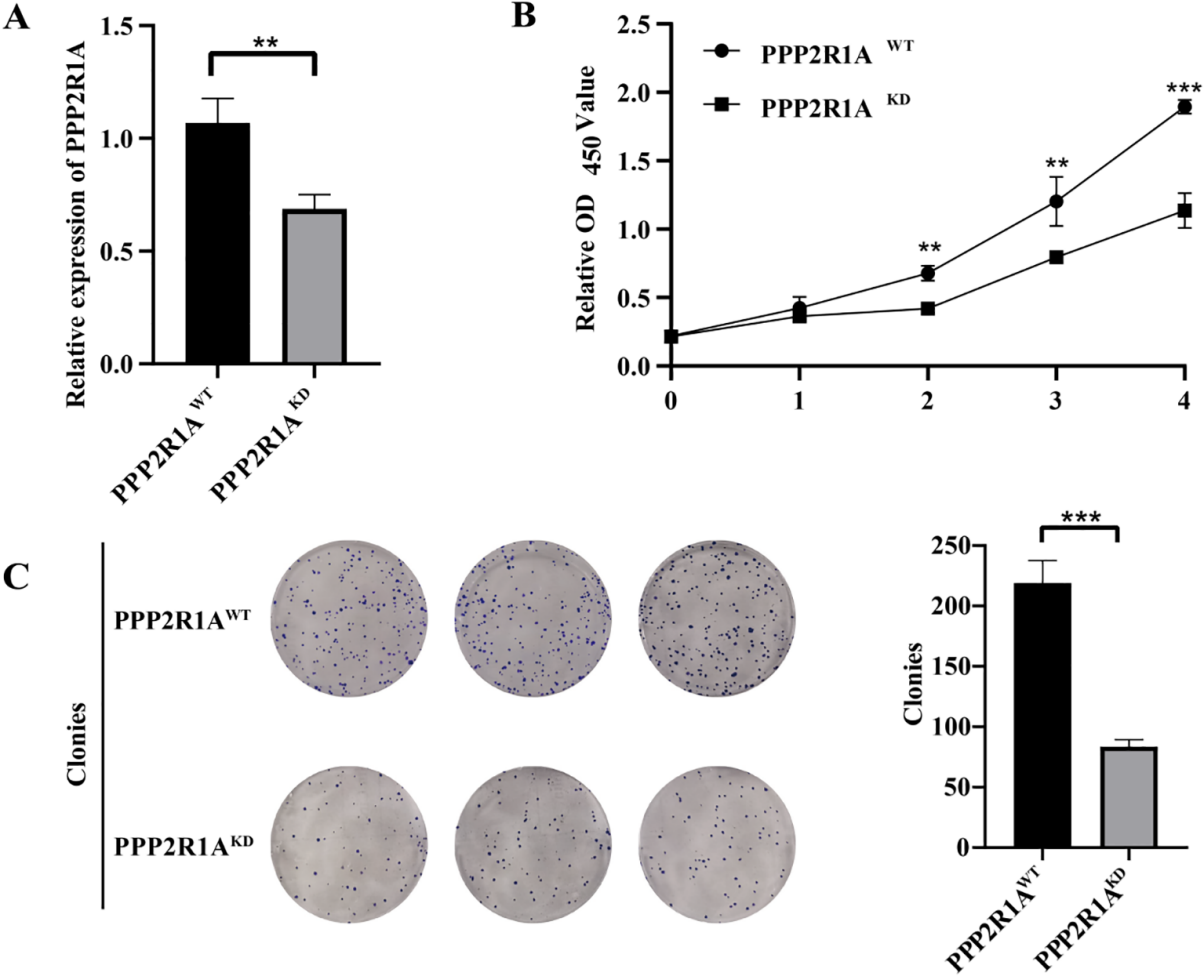
The knockdown of PPP2R1A reduces the proliferation and clonogenic potential of the A549 cell line. **(A)** Transcriptional silencing efficacy was quantified by RT-qPCR. **(B)** CCK-8 assay. **(C)** Clonogenic assay. (n=3) **p < 0.01, ***p < 0.001.

Based on bioinformatics predictions, PPP2R1A is closely linked to the progression of lung adenocarcinoma. To further explore its role in LUAD, this study performed cellular experiments for validation. qPCR analysis revealed a significant reduction in mRNA expression levels in the PPP2R1A knockdown group (*P* < 0.01), confirming the efficiency of gene knockdown (**Figure 10A**). CCK-8 assays revealed that the relative OD450 values in the PPP2R1A knockdown (KD) group were significantly lower than those in the wild-type (WT) group at 48, 72, and 96 hours. The fold changes (KD/WT) at 0, 24, 48, 72, and 96 hours were 1.01, 1.17, 1.62, 1.51, and 1.67, respectively (all P < 0.05 after 24 hours) (**Figure 10B**). Clonogenic assays demonstrated that PPP2R1A knockdown severely impaired colony formation, with the KD group forming significantly fewer visible colonies than the WT control group (fold change = 2.62, *P* < 0.001) (**Figure 10C**). All quantitative data are now presented as mean ± SD from n=3 independent experiments.

**Figure 11:**
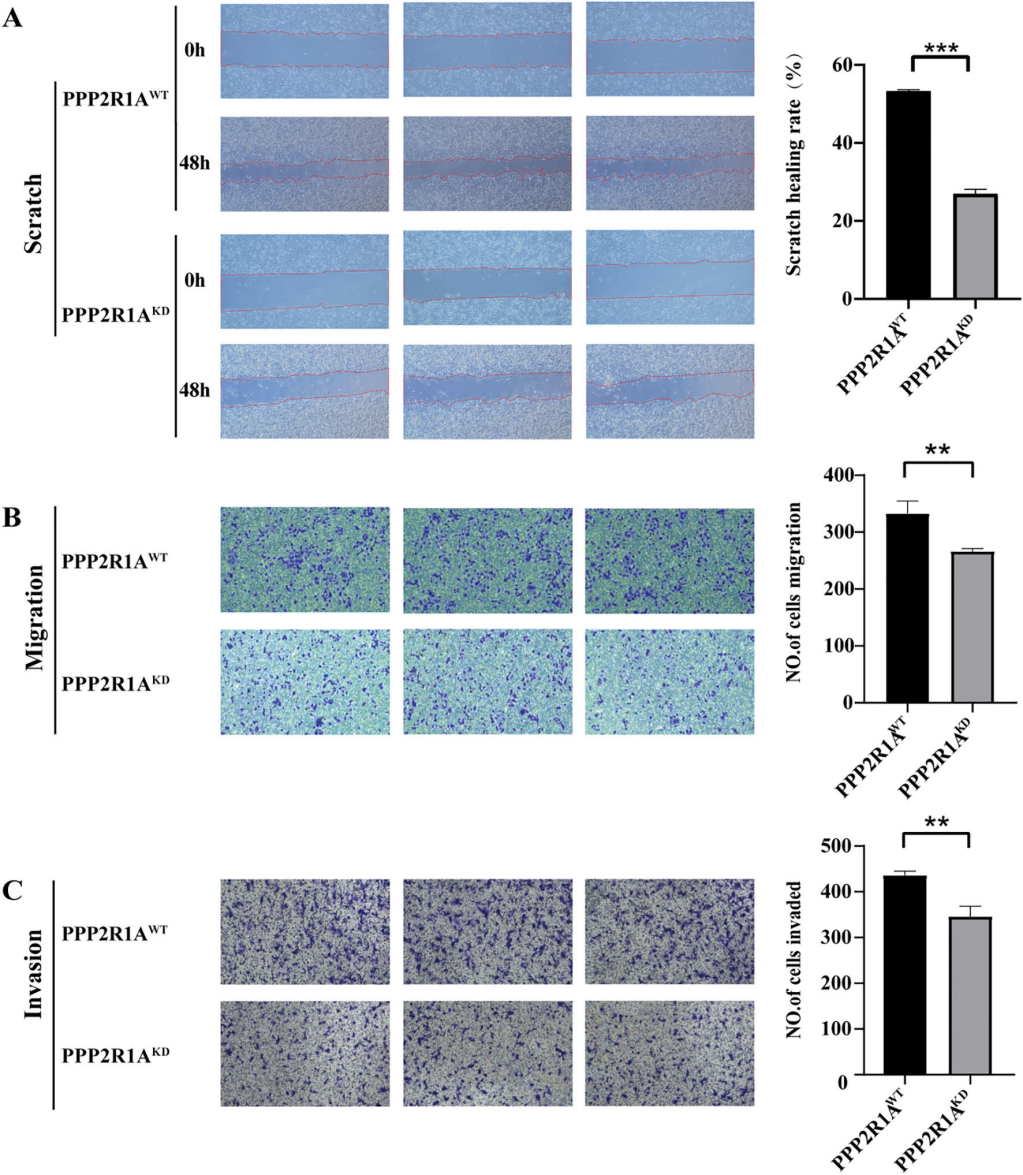
The knockdown of PPP2R1A inhibited the migratory and invasive phenotypes of A549 cells. **(A)** Wound healing assay. **(B)** Transwell migration assay. **(C)** Matrigel-invasive assay. WT (wild-type) cells; KD, PPP2R1A knockdown cells. (n=3) **p < 0.01, ***p < 0.001.

Cell scratch assays revealed a notable impairment in wound closure in the KD group compared to the WT group at the 48-hour time point (fold change = 1.99, *P* < 0.001) (**Figure 11A**). Transwell migration assays showed a significant decrease in matrix-penetrating cells in the PPP2R1A-KD group, indicating reduced migratory capacity (fold change = 1.25, *P* < 0.01) (**Figure 11B**). Additionally, the matrix gel invasion assay confirmed that PPP2R1A knockdown led to a significant reduction in the number of invasive cells (fold change = 1.26, *P* < 0.01) (**Figure 11C**). All representative images have been replaced with higher-resolution versions, and quantitative data (fold changes, exact p-values) are now explicitly stated in the figure legends and main text.

## Discussion

This study unveils the dual role of PPP2R1A in lung adenocarcinoma (LUAD) through a multidimensional approach: it serves as both a molecular driver of tumor progression and a key regulator of the immune microenvironment. Previous studies have found that PPP2R1A acts as a tumor suppressor gene in various cancers (30, 31). However, our analysis in lung adenocarcinoma reveals that it functions as an oncogene, suggesting that PPP2R1A is subject to complex regulation in tumors. Its role may vary across different cancers or under different external conditions, where it may exert distinct functions.

As a core scaffold subunit of the PP2A complex and a regulator of migration persistence (32), the function of PPP2R1A has traditionally been thought to inhibit pro-tumor signaling pathways (PI3K/AKT and MAPK) through dephosphorylation (33, 34). However, this study identifies PPP2R1A as a novel prognostic biomarker in LUAD, with its overexpression significantly correlating with reduced overall survival and progression-free survival. This paradoxical observation may arise from overexpression leading to excessive regulation of these signaling proteins, resulting in cell cycle dysregulation and promoting abnormal cell proliferation (35). Alternatively, this could be attributed to mutation-driven gain-of-function alterations and involvement in cell proliferation and migration processes, thereby influencing tumor progression (36). PPP2R1A consists of 15 HEAT repeat sequences (37), and its HEAT_2 domain harbors a critical missense mutation (Q372L) that is essential for the assembly of the PP2A holoenzyme. This mutation disrupts complex stability, thereby reducing phosphatase activity and enhancing oncogenic signaling (e.g., PI3K-AKT-mTOR) (38). Similar mechanisms have been reported in other cancers: in uterine cancer, PPP2R1A mutations exert a dominant-negative effect by binding to TIPRL1, inhibiting PP2A activity, and activating oncogenic pathways such as AKT to promote malignant growth (39). Although PPP2R1A mutation frequency is low (2.3%) in LUAD, its overexpression can induce functional dysfunction via a dose-response effect, suggesting that gene amplification or transcriptional regulation abnormalities may have greater clinical significance than mutations. Our knockdown experiments further support this hypothesis: PPP2R1A knockdown significantly inhibited the proliferation (*P* < 0.01), migration (*P* < 0.001), and invasion capacity (*P* < 0.01) of A549 cells.

To elucidate the functional role of PPP2R1A in LUAD pathogenesis, we performed gene identification and GO and KEGG enrichment analyses of PPP2R1A-related genes. PPP2R1A exhibited strong co-expression relationships with PRPF31 (R = 0.72) and SCAF1 (R = 0.72), suggesting its potential involvement in processes beyond traditional phosphatase activity, such as RNA splicing regulation. PRPF31 is a core component of the spliceosome (40), and its abnormal expression may lead to the generation of oncogenic isoforms (e.g., BCL-XL) (41). Based on the GO enrichment result for the ‘snRNA metabolic process’, PPP2R1A may indirectly influence the alternative splicing of tumor-related genes by regulating the activity of splicing factors. Overexpression of PPP2R1A could maintain high levels of BCL-XL expression by stabilizing the PRPF1 complex, thereby inhibiting tumor cell apoptosis. This hypothesis is further supported by the significance of the ‘cellular senescence’ pathway in KEGG enrichment analysis, as BCL-XL overexpression has been shown to bypass the senescence barrier and promote tumor progression (42). However, this proposed mechanism requires direct experimental validation in future studies.

Kaplan-Meier (KM) analysis indicates that LUAD patients with high PPP2R1A expression experience significantly worse clinical outcomes. Our immune infiltration analysis revealed a more complex and unexpected relationship between PPP2R1A and the tumor immune microenvironment. Contrary to the typical M2-skewed immunosuppressive profile often associated with poor prognosis, PPP2R1A expression showed predominantly negative correlations with the majority of both M1 and M2 macrophage polarization markers. The most notable exception was a strong positive correlation with TGFB1, a potent immunosuppressive cytokine. This unique signature, characterized by a TGFB1-high state alongside general downregulation of other macrophage markers, may represent a distinct immunosuppressive niche. TGF-β is a master regulator of immune suppression, known to inhibit cytotoxic T cell and NK cell function while promoting the generation of induced Tregs (43). Thus, the poor prognosis in patients with high PPP2R1A may be primarily driven by this dominant TGF-β signaling axis, which overrides the need for full classical M2 polarization.

Similarly, the relationship with regulatory T cells (Tregs) presented a paradox. While PPP2R1A did not correlate with the Treg lineage-defining transcription factor FOXP3, it showed significant negative correlations with key functional effector molecules such as CTLA4 and CCR8. This dissociation between lineage presence and functional marker expression is intriguing. One plausible explanation is that PPP2R1A is associated with a specific subset of Tregs that are FOXP3+ but have an altered functional or metabolic state, leading to reduced expression of classic checkpoint molecules. Alternatively, high PPP2R1A might create a microenvironment where the constitutive immunosuppressive pressure is so high, potentially via the TGF-β pathway (44), that Tregs do not need to upregulate effector molecules like CTLA4 to maintain suppression. This hypothesis is consistent with the established role of PP2A in fine-tuning T cell receptor signaling and cellular metabolism (32), which could lead to altered Treg functionality.

In a stratified Cox regression analysis of the PPP2R1A gene and LUAD, high PPP2R1A expression was found to lead to the enrichment of regulatory T cells (Tregs), resulting in poorer patient prognosis. Notably, high Treg infiltration has been linked to poor outcomes in various cancers (45), consistent with our findings.

The clinical translational potential of this study is twofold: first, PPP2R1A expression levels can serve as a prognostic biomarker for LUAD stratification, particularly for early-stage (T1) patients, where high expression correlates with significantly shorter progression-free survival (FP), suggesting these patients may benefit from more aggressive adjuvant therapy. Second, small-molecule stabilizers targeting the PPP2R1A-PP2Ac complex interface (e.g., DT-061) have demonstrated the potential to restore PP2A’s tumor-suppressing function in various cancer models, including lung and breast cancer (46). This provides new insights for targeted therapies in LUAD.

However, this study has several limitations. First, the diagnostic value of PPP2R1A mRNA alone is modest (AUC = 0.593), limiting its utility as a standalone diagnostic marker. We identified a discrepancy in PPP2R1A expression trends between TCGA (mRNA upregulation) and CPTAC (mRNA and protein downregulation) datasets. Our analysis revealed a significant negative correlation (r = -0.5090) between PPP2R1A protein abundance and mRNA expression in tumor and adjacent non-tumor tissues. This suggests potential post-transcriptional regulation of PPP2R1A during tumorigenesis. The observed differences highlight the influence of patient cohorts, sample processing, and normalization methods on gene expression analysis. Further studies using standardized assays on well-characterized patient samples are warranted to elucidate PPP2R1A’s role in cancer. It suggests that PPP2R1A’s role may be context-dependent, and future studies should validate its expression in well-characterized, matched patient samples using standardized assays.

Second, our immune infiltration analysis relied on computational algorithms. Experimental validation using techniques such as immunohistochemistry (IHC), flow cytometry, or single-cell RNA sequencing (scRNA-seq) on LUAD patient tissues is needed to confirm these findings and precisely map PPP2R1A expression within specific cellular compartments of the tumor microenvironment. Third, the experimental validation was conducted in a single LUAD cell line (A549, KRAS-mutant). While the stable CRISPR/Cas9-mediated knockdown model strengthens our conclusions, future work should validate these functional roles in additional LUAD cell lines (e.g., H1975, PC9) and normal lung cell lines to ensure generalizability. Furthermore, rescue experiments and investigation into specific downstream mechanisms, such as direct assessment of PD-L1 or other immune checkpoint molecules upon PPP2R1A modulation, are important next steps. Finally, the intriguing co-expression with splicing factors like PRPF31 warrants direct experimental investigation to establish a causal link between PPP2R1A and RNA splicing dysregulation in LUAD.

## Conclusion

We explored the pivotal role of PPP2R1A in LUAD through public gene expression database analysis and experimental validation in LUAD cell models. This study suggests that PPP2R1A is not only a potential diagnostic marker compared to existing markers but also a potential therapeutic target. Its knockdown significantly inhibits the malignant phenotypes of tumor cells. Moreover, we found that PPP2R1A expression can influence the tumor microenvironment by regulating immune cell infiltration, particularly by potentially promoting an M2-like macrophage polarization and Treg recruitment, thereby contributing to an immunosuppressive milieu. Despite the limitations, our findings provide a foundation for future research into the multifaceted roles of PPP2R1A in LUAD pathogenesis and immunotherapy.

## Data Availability

The original contributions presented in the study are included in the article/**Supplementary Material**. Further inquiries can be directed to the corresponding author/s.

## Supplementary material

Supplementary Figure 1 Differential Expression of PPP2R1A in LUAD Tumor and Adjacent Normal Tissues at Protein and mRNA Levels. **(A)** Relative protein abundance. **(B)** mRNA expression counts. **(C)** Protein to mRNA Level Correlation. ****p* < 0.001.

Supplementary Figure 2 Correlation Analysis of PPP2R1A Expression with Markers of M1/M2 Macrophages and Regulatory T Cells (Tregs) in Lung Adenocarcinoma. **(A)** Heatmap showing the correlation between PPP2R1A expression and canonical markers for M1 macrophages (NOS2, IL6, TNF, CD86, CD80) in the TCGA-LUAD cohort. PPP2R1A demonstrates weak negative or non-significant correlations with M1 markers. **(B)** Heatmap showing the correlation between PPP2R1A expression and canonical markers for M2 macrophages (IL10, ARG1, MRC1, CD163, TGFB1). PPP2R1A shows significant positive correlations with key M2 markers, including ARG1, CD163, MRC1 (CD206), IL10 and TGFB1. **(C)** Heatmap showing the correlation between PPP2R1A expression and key Treg markers (CTLA4, CCR8, FOXP3 and IL2RA). PPP2R1A expression is significantly positively correlated with both markers.
