# Supplementary figures and images for: Expression characteristics, prognostic value, and immune-related analysis of PPP2R1A in lung adenocarcinoma
Source: Front Immunol. 2025 Dec 2;16:1652629. doi: 10.3389/fimmu.2025.1652629 (PMC12705640; doi:10.3389/fimmu.2025.1652629)

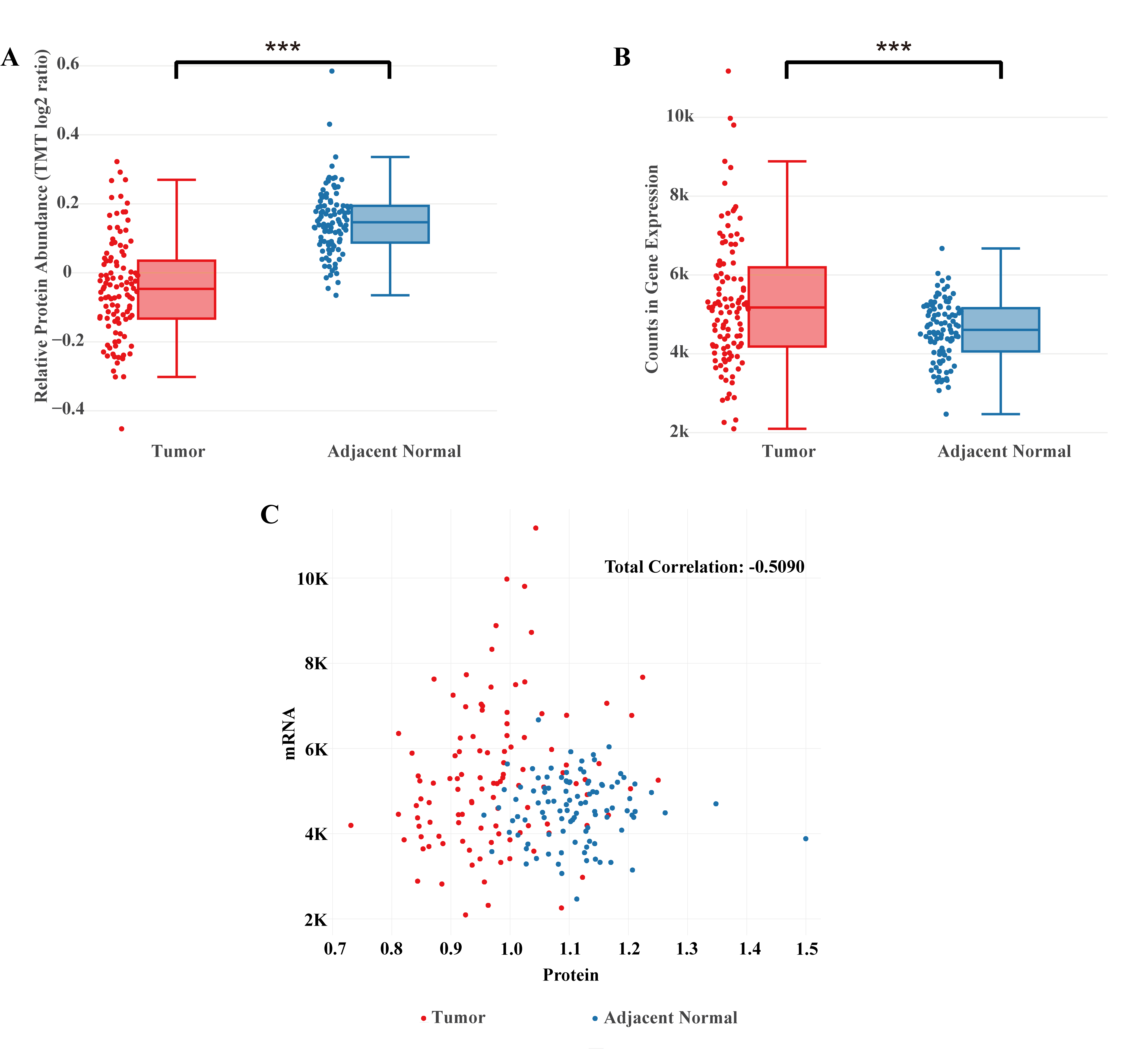

Supplement: Supplementary Figure 1 — Differential Expression of PPP2R1A in LUAD Tumor and Adjacent Normal Tissues at Protein and mRNA Levels. (A) Relative protein abundance. (B) mRNA expression counts. (C) Protein to mRNA Level Correlation. ***p < 0.001. [file Image1.png]

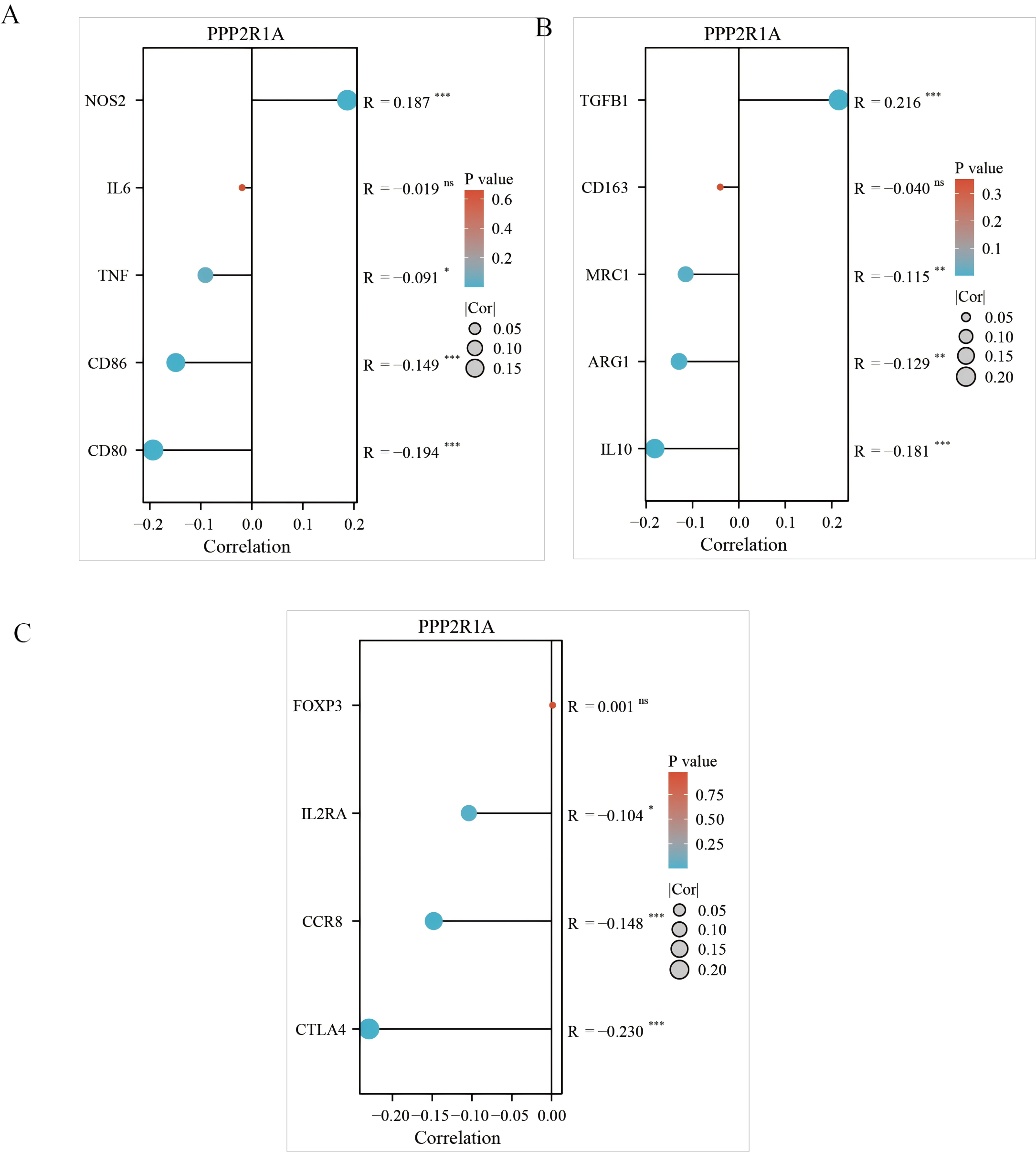

Supplement: Supplementary Figure 2 — Correlation Analysis of PPP2R1A Expression with Markers of M1/M2 Macrophages and Regulatory T Cells (Tregs) in Lung Adenocarcinoma. (A) Heatmap showing the correlation between PPP2R1A expression and canonical markers for M1 macrophages (NOS2, IL6, TNF, CD86, CD80) in the TCGA-LUAD cohort. PPP2R1A demonstrates weak negative or non-significant correlations with M1 markers. (B) Heatmap showing the correlation between PPP2R1A expression and canonical markers for M2 macrophages (IL10, ARG1, MRC1, CD163, TGFB1). PPP2R1A shows significant positive correlations with key M2 markers, including ARG1, CD163, MRC1 (CD206), IL10 and TGFB1. (C) Heatmap showing the correlation between PPP2R1A expression and key Treg markers (CTLA4, CCR8, FOXP3 and IL2RA). PPP2R1A expression is significantly positively correlated with both markers. [file Image2.png]
